# Fluorescence detection of pituitary neuroendocrine tumour during endoscopic transsphenoidal surgery using bevacizumab-800CW: a non-randomised, non-blinded, single centre feasibility and dose finding trial [DEPARTURE trial]

**DOI:** 10.1007/s00259-024-06947-9

**Published:** 2024-10-11

**Authors:** I. Schmidt, R. A. Vergeer, M. R. Postma, G. van den Berg, A. J. Sterkenburg, A. G. W. Korsten-Meijer, R. A. Feijen, S. Kruijff, A. P. van Beek, W. F. A. den Dunnen, D. J. Robinson, J. M. C. van Dijk, W. B. Nagengast, J. M. A. Kuijlen

**Affiliations:** 1 Department of Gastroenterology and Hepatology, University of Groningen, University Medical Center Groningen, Groningen, The Netherlands; 2 Department of Neurosurgery, University of Groningen, University Medical Center Groningen, P.O. Box 30.001 (AB-71), Groningen, 9700 RB The Netherlands; 3 Department of Endocrinology, University of Groningen, University Medical Center Groningen, Groningen, The Netherlands; 4 Department of Otorhinolaryngology– Head and Neck Surgery, University of Groningen, University Medical Center Groningen, Groningen, The Netherlands; 5 Department of Surgical Oncology, University of Groningen, University Medical Center Groningen, Groningen, The Netherlands; 6 Department of Pathology, University of Groningen, University Medical Center Groningen, Groningen, The Netherlands; 7 Center for Optical Diagnostics and Therapy, Department of Otorhinolaryngology– Head and Neck Surgery, Erasmus MC Cancer Institute, Rotterdam, The Netherlands

**Keywords:** Quantitative fluorescence molecular endoscopy, Pituitary neuroendocrine tumor, Bevacizumab, Transsfenoidal surgery

## Abstract

**Purpose:**

Achieving endocrine remission by gross total resection is challenging in pituitary neuroendocrine tumours (PitNETs) with cavernous sinus invasion. This study aims to assess the safety, feasibility, and optimal dose for intraoperative fluorescence imaging as an added instrument to discriminate PitNET from surrounding tissue using bevacizumab-800CW, targeting vascular endothelial growth factor A (VEGF-A).

**Methods:**

In part I, dose-escalation (0–4∙5-10-25 mg) was performed in 4 groups of 3 patients with PitNETs Knosp grade 3–4. In part II, after interim analysis, the 10 mg and 25 mg groups were expanded to a total of 6 patients. Quantitative fluoroscence molecular endoscopy consisted of wide field fluorescence molecular endoscopy and multi-diameter single fiber reflectance / single fiber fluorescence spectroscopy. Mean fluorescence intensity (MFI) of the fresh surgical specimen was calculated and VEGF-staining was performed.

**Results:**

Eighteen patients were included. All doses were well tolerated. Three serious adverse events were registered, but none were tracer-related. Part I showed an adequate in-vivo tumour-to-background ratio for both 10 mg (TBR 2∙00 [1∙86, 2∙19]) and 25 mg (TBR 2∙10, [1∙86, 2∙58]). Part II revealed a substantially higher MFI in the 25 mg group. With both 10 mg and 25 mg a statistically significant difference between tumour and surrounding tissue was detected (*p* < 0∙0001). All surgical specimens had VEGF-A expression.

**Conclusion:**

This study demonstrates the safety and feasibility of quantitative fluorescence molecular endoscopy during PitNET surgery. Both 10 mg and 25 mg bevacizumab-800CW result in clear differentiation in-vivo, with improved contrast ex-vivo (MFI) in the 25 mg group.

**Trial registration:**

NCT 04212793 / Study Details| Detection of PitNET Tissue During TSS Using Bevacizumab800CW| ClinicalTrials.gov.

**Supplementary Information:**

The online version contains supplementary material available at 10.1007/s00259-024-06947-9.

## Introduction

Pituitary neuroendocrine tumours (PitNETs) are classified based on hormone production, histology, size, and invasiveness. Transsphenoidal surgery (TSS) is the primary treatment of choice for most PitNETs, except for lactotroph tumours. The objective of surgery is to decompress vital structures and to achieve endocrine remission (ER). Gross total resection (GTR) is related to tumour invasiveness, which is categorized using the Knosp classification. Knosp grade 3 or 4 indicates cavernous sinus (CS) invasion and thus limits GTR and ER. For clinically non-functioning PitNETs the rate of GTR in Knosp 3 and 4 is 67% and 0% respectively [1–3]. For somatotroph PitNETs the pooled overall surgical remission rate according to the 2010 criteria for all included papers was 54.8% (95% CI 44.4–65.2%) for macroadenomas, 29% (95% CI 20.1–37.8%) for invasive adenomas, and 68.8% (95% CI 60-77.6%) for non-invasive adenomas [4]. This can be explained by an increased rate of tumour invasion into the medial wall of the cavernous sinus [5]. 

Surgeons rely on visual and tactile information to differentiate between tumour and normal surrounding tissue. Given that endoscopic visualization of the inferior CS compartment is difficult, an additional instrument to differentiate between residual tumour and surrounding tissue may improve GTR and prevent recurrent disease. As such, this addition could limit morbidity and substantially reduce lifetime treatment costs [6]. 

Fluorescence-guided molecular imaging provides real-time information during surgery, using tracers that target specific proteins. In PitNET surgery, 5-ALA, ICG, OTL38, and fluorescein were evaluated previously using commercially available imaging systems [7–9]. These studies often only used semi-quantitative data, e.g., tumour-to-background ratio (TBR), which are influenced by optical tissue properties such as absorption and scattering, and imaging conditions like distance and angle. Hence, reproducibility and patient-to-patient comparison were complicated [10–12]. In this study, we evaluated quantitative fluorescence molecular endoscopy (qFME) for distinction between PitNET and normal surrounding tissue. qFME combines fluorescence molecular endoscopy (FME) with multidiameter single-fiber reflectance / single-fiber fluorescence (MDSFR/SFF) spectroscopy. The latter enables determination of intrinsic fluorescence by correcting for tissue absorption and scattering. Bevacizumab-800CW targets vascular endothelial growth factor A (VEGF-A) and is a promising tracer, since it is overexpressed in PitNETs with CS invasion [13], lactotroph PitNETs, and pituitary carcinomas [14–17]. Previously, the combination of qFME and bevacizumab-800CW has been successfully applied in gastroenterological endoscopy and surgery to differentiate between tumour and normal tissue [18, 19]. 

The aim of this study is to assess the safety, feasibility, and most optimal dose of qFME with bevacizumab-800CW to identify PitNET during endoscopic TSS.

## Methods

### Study design

This study was performed at the department of Neurosurgery of the University Medical Center Groningen (UMCG) in The Netherlands. Adult patients (≥ 18 years) with a performance status of WHO 0–2 and scheduled TSS of a PitNET Knosp grade 3–4 were included. Patients were informed about the aim of the study and study-related procedures and written informed consent was obtained. The study was approved by the institutional review board (METc 2019/713) and registered in ClinicalTrials.gov (NCT 04212793). A substantial amendment was added to the original protocol to include a control group.

### Quantitative fluorescence molecular endoscopy

Bevacizumab-800CW was intravenously administered three days prior to TSS. During surgery, qFME was performed at predefined stages: (1) before opening of the dura; (2) after dural opening, with meaningful tumour-quantity in sight; (3) after removal of tumour from the sella; (4) during inspection of the medial wall of the cavernous sinus. The inspection before dural opening (step 1) was added after publication of the original protocol [20]. Pituitary tissue was considered optimal background tissue but was not available in all patients. Therefore, nasal mucosa measurements were added to the protocol after the second procedure and used as a non-tumourous background signal.

**Fig. 1:**
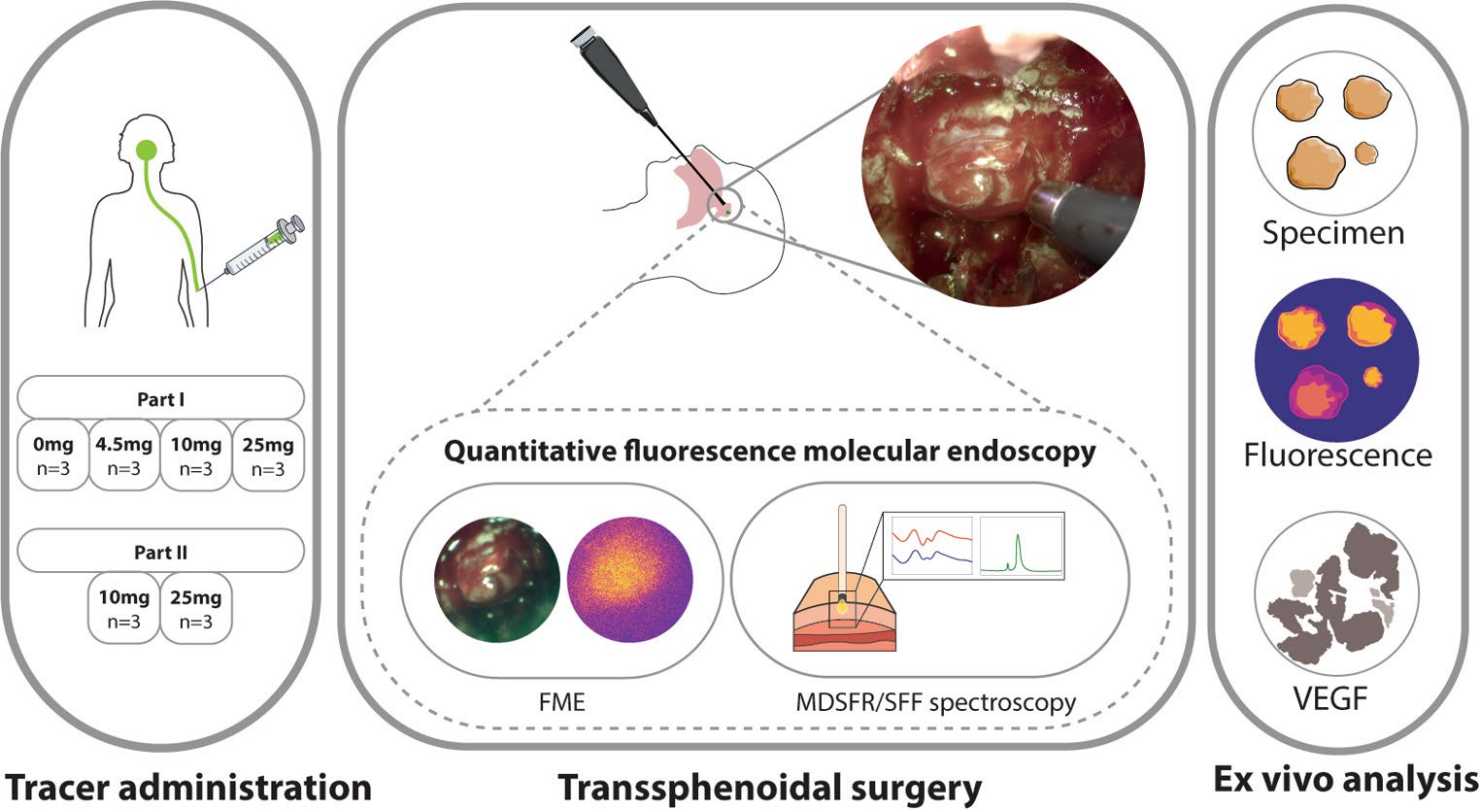
Overview of study design. (**A**) Tracer administration was performed 3 days before transsphenoidal surgery with 4 different tracer doses according to protocol. (**B**) During transsphenoidal surgery qFME was performed consisting of FME and MDSFR/SFF spectroscopy. MDSFR/SFF spectroscopy quantifies the fluorescence signal by performing reflectance measurements with two different diameters (red and blue) and correcting the raw fluorescence (green) for the optical properties determined from the reflectance spectra. (**C**) Ex-vivo analyses were performed to validate the in-vivo results. qFME = quantitative fluorescence molecular endoscopy, FME = fluorescence molecular endoscopy, MDSFR/SFF = multidiameter single-fiber reflectance / single fiber fluorescence

FME was performed with a fiber-based fluorescence endoscopic camera (SurgVision, Bracco), with excitation at 750 nm. The fiber bundle with a field-of-view of 85 degrees was directly inserted through the nose, guided by the white light endoscopy camera. The system enabled a simultaneous view of the white light, fluorescence, and overlay image. Subsequently, MDSFR/SFF spectroscopy was performed to quantify the in-vivo fluorescence signal. The spectroscopy fiber was inserted through a rigid suction tube to stabilize measurements that were taken by gently pushing the fiber against nasal mucosa, PitNET, pituitary, and the wall of the cavernous sinus. Reflectance spectra with two different diameters were taken, followed by a fluorescence spectrum with the larger of the two diameters. By using two fiber diameters the tissue optical properties (absorption, µ_a_ and scattering, $\:{\mu\:}_{s}^{{\prime\:}}$) were determined. The raw fluorescence signal was corrected for these optical properties to obtain quantitative intrinsic fluorescence [21–23] (See Fig. 1).

### Safety

Vital signs (temperature, heart rate and blood pressure) were monitored from before administration until 1 h after administration of Bevacizumab-800CW. (Serious) adverse event ((S)AE) assessment was performed according to the National Cancer Institure Common Terminology Criteria for Adverse Events (CTCAE) version 5∙0. Serious adverse events, if present, were reported to the Dutch central committee on research involving human subjects (CCMO). SAEs were followed until fully resolved or a stable situation was achieved.

### Dose escalation

**Fig. 2:**
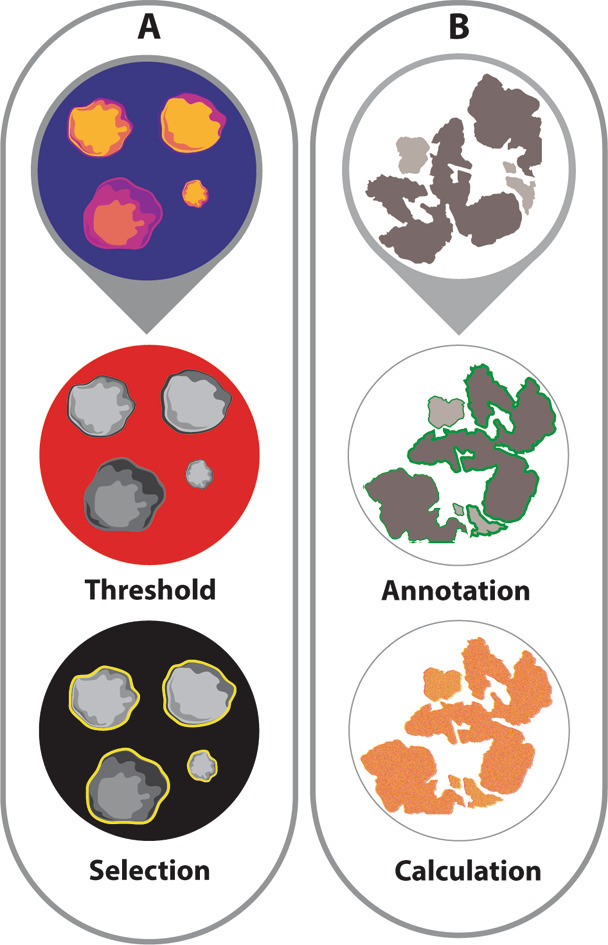
Methods for ex-vivo validation. (**A**) MFI calculation based on the fresh surgical specimen. A threshold is applied to the Odyssey images and the MFI is calculated on the selected area (**B**) The 4 μm VEGF-A-stained slice is annotated and the VEGF-A expression is calculated per annotation

A two-part dose escalation study was performed. In part 1, dose escalation (0–4∙5-10-25 mg) was performed in 4 groups of 3 patients. These doses were based on previous trials [20, 24]. An interim analysis was performed on both in-vivo and ex-vivo data. This analysis consisted of evaluation of the quantitative fluorescence signal based on MDSFR/SFF spectroscopy and on MFI-values measured on the fresh surgical specimen, using an Odyssey CLx flatbed scanner (LI-COR Biosciences Inc., Lincoln, NE, USA). A tumour-to-background ratio was calculated using the intrinsic fluorescence. MFI was calculated by applying a threshold on fluorescence imaging and measuring MFI only within the selected area with ImageJ Fiji (version 2∙9∙0 / 1∙53) (Fig. 2). Depending on interim analysis results, three scenarios were possible: (1) extension of one dose; (2) extension of two doses; (3) termination of the study. If two doses were extended, a final analysis would be necessary to define the optimal dose.

### Ex-vivo validation

All tumour fragments were sent to Pathology for processing into formalin-fixated and paraffin-embedded (FFPE) tissue blocks, cut into 4 μm slices for VEGF-A staining. VEGF-A staining (RB-9031-PO-A, Fisher scientific) was analyzed with the positive pixel count v9 Aperio ImageScope (version 12∙4∙3∙5008, Leica Biosystems Imaging Inc, Vista, USA). Annotations were manually drawn on each slide to identify the region of interest for analysis. The algorithm output was composed of the number of strong positive pixels (Nsp), the number of weak positive pixels (Nwp), and total number of pixels (NTotal). A value for VEGF-A staining was obtained for each annotation:

$$VEGFA\,expression\left( {H - score} \right) = \frac{{\left( {1*Nwp} \right) + \left( {2*Nsp} \right)}}{{NTotal}}$$

### Statistical analysis

Descriptive statistics were performed on patient demographics. Given the small sample size, all data were considered non-normally distributed. A tumour-to-background ratio (TBR), based on spectroscopy measurements, was calculated for all patients by dividing the weighted mean of the tumour by the weighted mean of the normal tissue. Data was presented as median with interquartile range (IQR). Differences between unpaired data were tested using Mann-Whitney. For comparison between multiple groups a one-way ANOVA was used for normally distributed data and a Kruskal-Wallis test for skewed data. Two-sided *p* < 0.05 was considered statistically significant. Statistical analysis and graph design were performed using GraphPad Prism (version 9∙4∙1, GraphPad Software Inc, San Diego, California, USA).

## Results

### Patient characteristics and safety

Between November 2021 and October 2022, twenty-one patients were included into four dose groups: 0 mg, 4∙5 mg, 10 mg, and 25 mg (Table 1). Three patients received 4∙5 mg, six patients 10 mg, six patients 25 mg, and three patients 0 mg (control group). Three patients were replaced: two patients (4∙5 mg and 10 mg) were excluded based on the histopathological diagnosis (mixed gangliocytoma-somatotroph adenoma and lymphangioma), and one patient (10 mg) had PitNET tissue that was too liquified to perform measurements and to acquire a surgical specimen. Residual tumour was found on MRI in 78% of patients (14 out of 18) at least 6 weeks after surgery. Administration of bevacizumab-800CW was considered safe in all dose cohorts. None of the registered (serious) adverse events were tracer-related. The three serious adverse events are further specified in Supplementary Table 1.

**Table 1:** Overview of patient characteristics

	0 mg *n* = 3	4.5 mg *n* = 3	10 mg *n* = 6	25 mg *n* = 6
Patient characteristics				
Sex				
Male	3	2	2	6
Female	0	1	4	0
Age, Median (quartiles)	58 (57–62)	70 (46–71)	59 (37–69)	65 (48–74)
Knosp grade				
3A	2	1	3	3
3B	0	0	0	3
4	1	2	3	0
Classification				
Gonadotroph	3	1	1	3
Null cell	0	1	0	1
Somatroph	0	0	2	0
Lactotroph	0	0	2	1
Corticotroph	0	1	1	1
Residual tumour				
Yes	3	3	4	4
No	0	0	2	2

### Dose escalation of bevacizumab-800CW

**Fig. 3:**
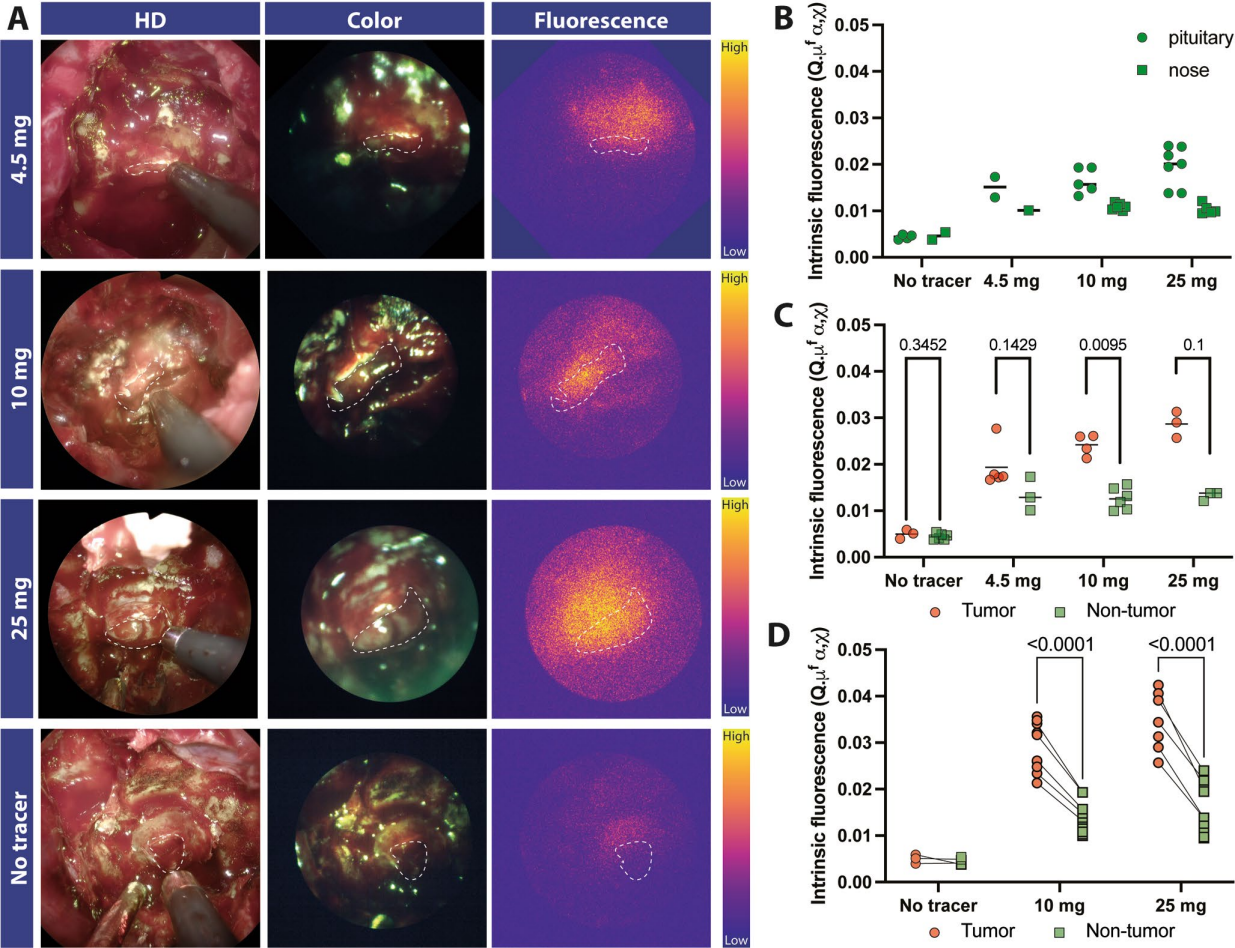
In-vivo dose-escalation results.(**A**) representative snapshots from exposed tumour (marked in white) from each dose cohort showing a clear difference compared to the control group. All images are scaled to each other (**B**) Pituitary and nose measurements which are combined and used as non-tumourous background tissue. (**C**) In-vivo intrinsic fluorescence values for both the tumour and non-tumourous tissue for part I of the dose-escalation. (**D**) Extension of the 10 and 25 mg groups in part II of the dose-escalation. Images were scaled to each other by stacking them and using a colour map (MPL-plasma)

Typical images of the dose groups are depicted (Fig. 3A). The second imaging timepoint ‘after dural opening, with meaningful tumour-quantity in sight’, was decided as the most valuable timepoint for the feasibility evaluation of fluorescence imaging as both the tumor and the background could be clearly visualized within the same image. All in vivo measurements described are performed during this timepoint.

**Fig. 4:**
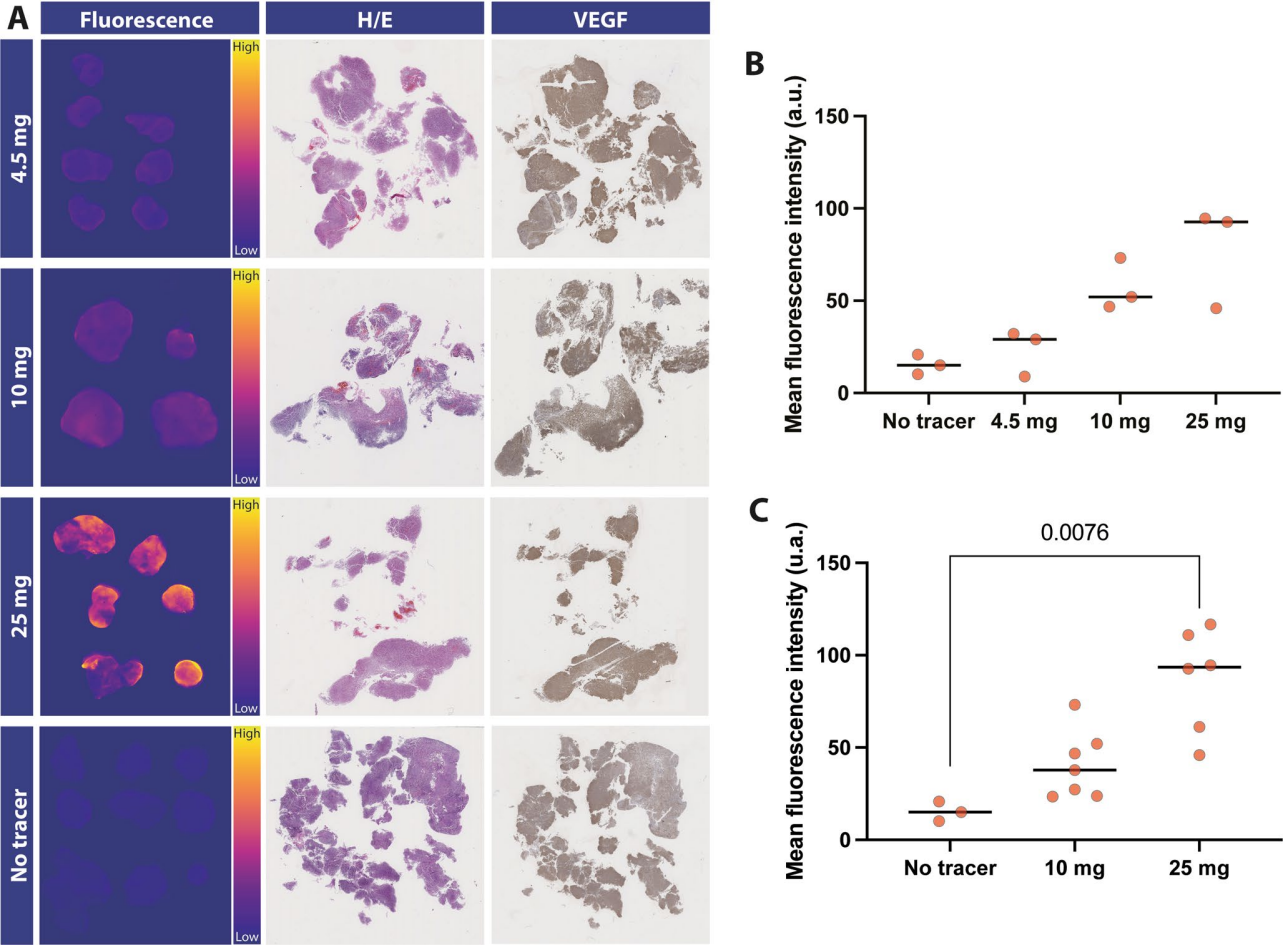
Ex-vivo dose-escalation results and VEGF staining. (**A**) Representative snapshots of the fresh surgical specimen are shown in the first column followed by the corresponding H/E and VEGF-A-stained tissue slices. (**B**) ex-vivo calculated MFI for part I of the dose-escalation and (**C**) part II of the dose-escalattion. H/E = Hematoxylin/ Eosin, VEGF-A = Vascular endothelial growth factor-A

A higher signal around the exposed tumour area (dotted white line) is noted in the 4∙5 mg, 10 mg, and 25 mg dose group, compared to the control group. Part I involved the 4 × 3 interim evaluation between 0 mg, 4∙5 mg, 10 mg, and 25 mg (Fig. 4C) using the intrinsic fluorescence by MDSFR/SFF spectroscopy. A dose-dependent increase in median value was measured with increasing doses (0 mg: 0∙005; 4.5 mg: 0∙017; 10 mg: 0∙025; 25 mg: 0∙029). In addition, the 10 mg and 25 mg groups showed the highest TBR (dose: [95% confidence interval]; 10 mg: 2∙00 [1∙86, 2∙19]; 25 mg: 2∙10, [1∙86, 2∙58]) compared to the lower dose groups (4∙5 mg: 1∙54, [1∙49, 1∙60]; 0 mg: 1∙17, [0∙896, 1∙45]*).* Representative images of the fluorescence within the fresh surgical specimen are shown in Fig. 4A. The mean fluorescence intensity, measured ex-vivo, also showed a dose-dependent increase (Fig. 4B-C). Based on interim analysis of in-vivo TBR and ex-vivo MFI, both the 10 mg and 25 mg groups were expanded to 6 patients. In part II, evaluating the expanded 10 mg and 25 mg groups, TBR remained comparable, with an increased significance between tumour and normal tissue (10 mg: 1∙99, [1∙66, 2∙53]; 25 mg: 2∙06, [1∙71, 3∙69]). Higher fluorescence values correlated with a higher value in non-tumourous tissue in both the 10 mg and 25 mg groups (Fig. 4D). Ex-vivo, both dose groups showed a statistically significant difference compared to the control group, with the highest discrimination between tumour and normal surrounding tissue of all doses studied in the 25 mg group.

### Ex-vivo validation of VEGF-A expression

Corresponding Hematoxylin/Eosin (H/E) and VEGF-A images are depicted for each dose-group. Calculated mean VEGF-A value ranged between 0.92 and 1.4, indicating substantial VEGF-A expression in all slides. The lowest VEGF-A value in an individual annotation was 0.81; the highest was 1.4 (Supplementary Fig. 1). No differences in expression were observed between functioning and non-functioning tumours and between tumour types.

## Discussion

In this non-randomised, non-blinded, single centre, feasibility and dose-finding trial, we demonstrated the safety and feasibility of quantitative fluorescence molecular endoscopy (qFME) during PitNET surgery using bevacizumab-800CW, targeting VEGF-A. Both intravenous doses of 10 mg and 25 mg showed clear differentiation in-vivo, but 25 mg showed improved contrast compared to the control tissue (0 mg) in the ex-vivo measurements. Therefore, 25 mg was selected as the optimal dose of bevacizumab-800CW for the discrimination of PitNET from non-tumourous tissue.

In-vivo differentiation between tumour and surrounding tissue can be difficult, limiting GTR and ER. Highlighting the tumour during surgery using target tracers is evaluated in multiple fields to improve surgical resection rates [25–28]. In this study, we added MDSFR/SFF spectroscopy to the generally used wide field fluorescence imaging to provide objective quantitatitive fluorescence measurements. Here, the fluorescence is corrected for the scattering and absorption coefficient of the tissue, resulting in intrinsic fluorescence. Absorption and scattering both affect the measured fluorescence signal by decreasing and increasing the signal, respectively, which can hamper clinical decision making. Also, the fluorescence signal is influenced by factors such as ambient light, imaging distance, and angle [29]. Lee et al. used the distance between the two medial opticocarotid recesses as a landmark to estimate the distance between the camera and the tissue, to reduce the effect of distance on the fluorescence signal [10]. However, the fluorescence was still calculated using the subjective signal-to-background ratio. Our spectroscopy measurements were performed directly on the tissue, and therefore were not influenced by imaging distance or angle. This quantitative analysis of fluorescence is essential for an objective comparison in a dose finding study and in clinical use.

In this study, the tracer bevacizumab-800CW, targeting VEGF-A, was used. Although VEGF expression is known to vary between subtypes of PitNET, multiple studies showed moderate to high VEGF expression [17]. All patients in our study demonstrated high VEGF expression, strengthening our hypothesis that PitNET Knosp 3–4 patients could benefit from our new technique. The limited VEGF-A expression range (0.92–1.4) prevented correlation between the fluorescence intensity and VEGF-A expression in our study. Comparison between VEGF and fluorescence was further complicated by the limited range of VEGF expression between patients and the known mismatch between fluorescence intensity and immunohistochemistry. The polyclonal antibody used for our staining binds to only 3 of the 7 isoforms of bevacizumab, isoform 121, 165, and 189, where bevacizumab-800CW can only bind to extracellular isoforms 165 and 189 instead of intracellular VEGF [30]. Therefore, a mismatch may arise between VEGF-A expression and measured fluorescence intensity [31]. However, several other studies have shown a clear correlation between bevacizumab uptake and VEGF expression [31, 32]. 

Based on the in-vivo FME images, discrimination between tumour and normal tissue was challenging. The small cavity and high power white light (used for guidance) induce reflections into fluorescence images. Although experienced users recognize this, less experienced users may not. Moreover, signal absorption is enhanced by the presence of blood, which is one of the main absorbers in tissue. Unfortunately, blood is abundantly present in the highly vascularized sellar region. If tumour tissue is covered with free blood, both spectroscopy measurements and fluorescence imaging are complicated. In this case, the fluorescence measured by spectroscopy is mainly related to the free blood instead of the underlying tumour tissue. Measurements with a high blood value within the absorption parameters were therefore excluded from analysis. Therefore, making the measurement area as blood free as possible is essential for accurate measurements, and thus for clinical decision making based on fluorescent measurements.

Quantitative fluorescence molecular endoscopy enables differentiation of tumour and non-tumourous tissue based on quantitative spectroscopy measurements using bevacizumab-800CW as a targeted tracer. However, to proceed to clinical implementation, visual guidance based on the fluorescence signal is necessary. This could help identify unanticipated tumour remnant at the end of a procedure and therefore increased GTR and ER rates. Additionally, the added value of endonasal resection of the medial wall of the CS on outcomes of pituitary surgery, especially in functioning PitNETs has been described [33]. qFME could be of use to identify tumour invasion in the medial wall of the CS and whether an additional resection of the medial wall is indicated. Hence, use of high-resolution images is important. Currently, a fiberscope is composed of 30.000 individual fibers, which limits the resolution and field of view. In addition, the fiber regularly fogs during its course through the nose, despite the use of anti-fog solution. Preferably, the fluorescence imaging system should be combined with a clinically used rigid endoscope, to increase resolution and field of view, as well as to enable fluorescence imaging. Based on the fluorescence guidance, additional spectroscopy measurements could be performed to have objective feedback on the intrinsic fluorescence signal. However, the analysis is performed after the procedure and real-time feedback is currently not available. With improved fiber configuration and faster analysis, it is possible to attain real-time feedback of fluorescence measurements in the near future. Future work should first focus on development of the technical aspects as described above and on real time analysis of the spectroscopy measurements. Subsequently, we intend to perform a phase II study to evaluate the clinical impact of fluorescence imaging using 25 mg bevacizumab-800CW, with larger cohorts classified based on histological subtype.

## Conclusion

In conclusion, this study demonstrates that an intravenous dose of 25 mg bevacizumab-800CW was clinically well tolerated and enabled the best discrimination between tumour and normal surrounding tissue of all doses studied. MDSFR/SFF spectroscopy allowed for objective, quantitative measurements of the fluorescence intensity in-vivo. Technical limitations, as described in the discussion, in both fluorescence molecular imaging and spectroscopy should be addressed before clinical implementation of fluorescence guided PitNET surgery is feasible.

## Electronic supplementary material

Below is the link to the electronic supplementary material.

Supplementary Material 1

## Data Availability

The datasets generated during and/or analysed during the current study are available upon reasonable request to the corresponding author. The data are not publicly available because of privacy regulations.
