# Supplementary figures and images for: Fluorescence detection of pituitary neuroendocrine tumour during endoscopic transsphenoidal surgery using bevacizumab-800CW: a non-randomised, non-blinded, single centre feasibility and dose finding trial [DEPARTURE trial]
Source: Eur J Nucl Med Mol Imaging. 2024 Oct 11;52(2):660–8. doi: 10.1007/s00259-024-06947-9 (PMC11732902; doi:10.1007/s00259-024-06947-9)

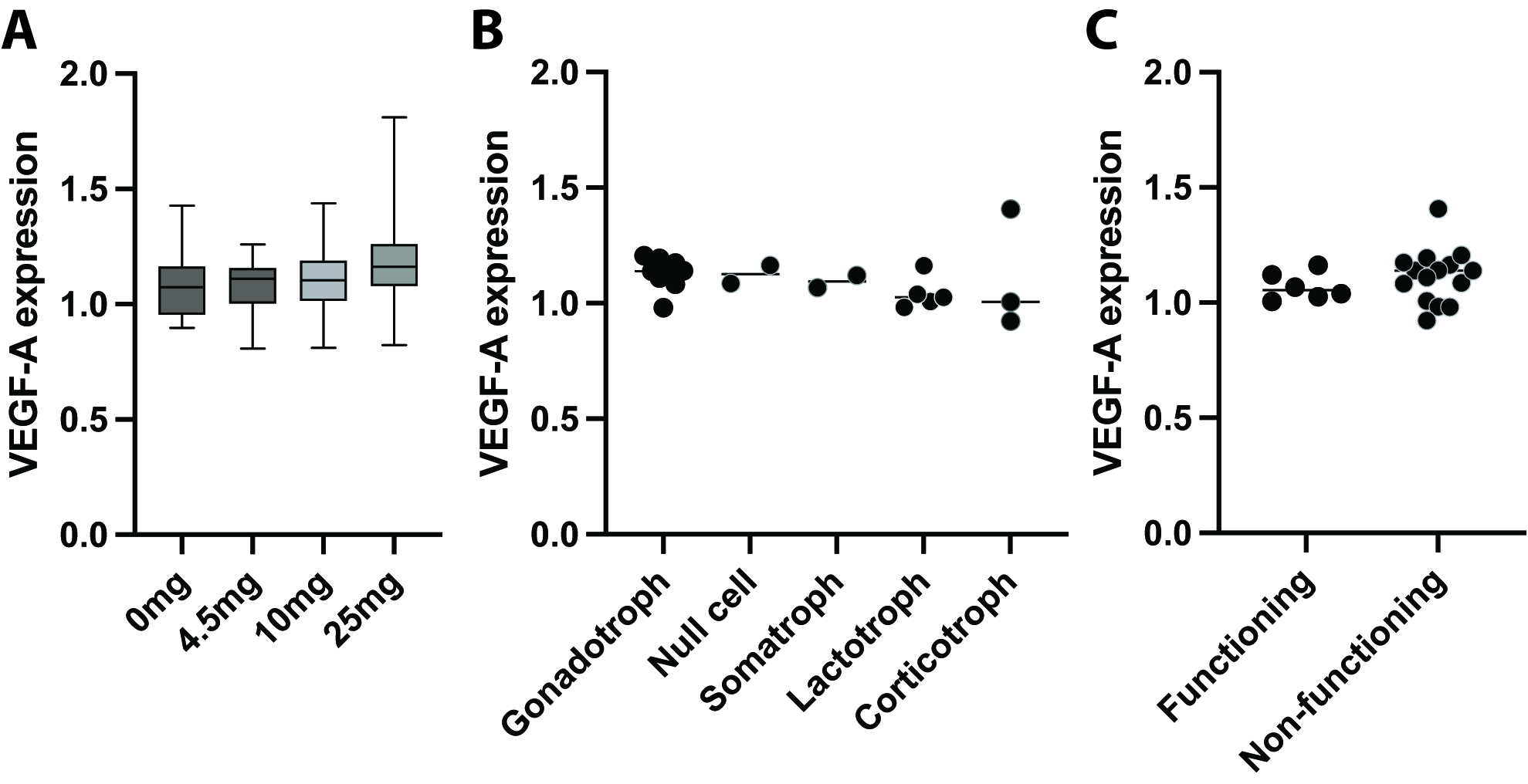

Supplement: Supplementary file 1 — Supplementary Material 1 [file 259_2024_6947_MOESM1_ESM.tif]
